# A system-oriented strategy to enhance electron production of *Synechocystis* sp. PCC6803 in bio-photovoltaic devices: experimental and modeling insights

**DOI:** 10.1038/s41598-021-91906-9

**Published:** 2021-06-10

**Authors:** Hossein Firoozabadi, Mohammad Mahdi Mardanpour, Ehsan Motamedian

**Affiliations:** 1 Department of Biotechnology, Faculty of Chemical Engineering, Tarbiat Modares University, P.O. Box 14115-143, Tehran, Iran; 2 Department of Chemical and Petroleum Engineering, Sharif University of Technology, Tehran, Iran

**Keywords:** Biotechnology, Systems biology

## Abstract

Bio-photovoltaic devices (BPVs) harness photosynthetic organisms to produce bioelectricity in an eco-friendly way. However, their low energy efficiency is still a challenge. A comprehension of metabolic constraints can result in finding strategies for efficiency enhancement. This study presents a systemic approach based on metabolic modeling to design a regulatory defined medium, reducing the intracellular constraints in bioelectricity generation of *Synechocystis* sp. PCC6803 through the cellular metabolism alteration. The approach identified key reactions that played a critical role in improving electricity generation in *Synechocystis* sp. PCC6803 by comparing multiple optimal solutions of minimal and maximal NADH generation using two criteria. Regulatory compounds, which controlled the enzyme activity of the key reactions, were obtained from the BRENDA database. The selected compounds were subsequently added to the culture media, and their effect on bioelectricity generation was experimentally assessed. The power density curves for different culture media showed the BPV fed by *Synechocystis* sp. PCC6803 suspension in BG-11 supplemented with NH_4_Cl achieved the maximum power density of 148.27 mW m^−2^. This produced power density was more than 40.5-fold of what was obtained for the BPV fed with cyanobacterial suspension in BG-11. The effect of the activators on BPV performance was also evaluated by comparing their overpotential, maximum produced power density, and biofilm morphology under different conditions. These findings demonstrated the crucial role of cellular metabolism in improving bioelectricity generation in BPVs.

## Introduction

Discovering the inherent capability of microorganisms to generate bioelectricity has opened a new era of renewable energy production. Regarding the emergence of microbial fuel cells (MFCs) as a system to extract electrons from the complex organic substrate during the anaerobic biodegradation process, the critical role of particular microorganisms as biocatalysts in wastewater treatment and energy generation has become crystal clear^1^. Although the MFCs are highly in academic interests^2^, the advantages of bio-photovoltaic devices (BPVs), which can operate without organic sources and carbon dioxide emission, reveal more benefits in comparison with MFCs encountering difficulties of supplying organic feed and carbon dioxide management^3^. In BPVs, the oxygenic photosynthetic organism is being used as a bio-anode catalyst, deriving electrons from water photolysis under the light emission^4^.

Despite the high potential of BPVs as a promising power generator, the main bottleneck restricting the commercial way is the intense competition of intracellular pathways for energy resources and intrinsic metabolic losses resulting in low power outputs generation^5^. Therefore, numerous researches were undertaken to understand the intracellular electron pathways of photosynthetic organisms. Among unicellular photosynthetic organisms, *Synechocystis* sp. PCC 6803 (henceforth referred to as *Synechocystis*), the well-known model organism, received a great deal of attention to investigate the electron transfer pathways^6^. In this regard, investigation of inhibitors' effects on the photosynthetic metabolic pathways^7^, creation of mutant strains to redirect electron flux^8^, and eliminating photosystem II^9^, has been demonstrated in previously published studies to understand electrons generation mechanism in *Synechocystis*.

Bombelli et al. investigated the use of classic inhibitors such as DCMU, DBMIB, and methyl viologen (MV), to identify the electron transfer chain suggesting that photo-electrons are originated from photolysis of water and end with the reducing form of PS I^7^. Bradley et al. redirected electron flux from competing electron sinks by creating mutant strains of *Synechocystis* lacking respiratory terminal oxidase complexes and using ferricyanide as an electron acceptor. When terminal oxidase complexes were inactivated, 10% of the respiratory electron flux was redirected to ferricyanide in the dark condition^8^. Cereda et al. studied the effect of photosystem II (PS II) on electron generation through both inhibitor addition and gene deletion. It was also proved that the primary photocurrent generation requires the activation of PS II during water photolysis^9^. Moreover, their case study on the *Synechocystis* revealed the incapability of transferring electrons to the electrode owing to numerous metabolic pathways facing fierce competition with the electrode for the electrons. Thus, extracellular photo-electrons are attributed to a small proportion of the total organism electron flux^9^. This matter has been pointed out in Glazier's work that cellular metabolism used only 3% of electrons produced by photosynthetic light reactions^10^, and thus the metabolism has considerable potential for electricity generation. However, switching the cellular metabolism to the generation of NADH is required.

Despite the careful investigations on the photosynthetic electron pathways, the importance of a system-oriented strategy applying metabolic modeling to uncover other metabolic pathways that may contribute to electrons production has been neglected. Mao and Verwoerd applied a metabolic network using flux balance analysis (FBA) to explore the inherent capacity of electron generation for *Synechocystis*; however, the lack of experimental works in their studies is evident^11^.

Enhancing intracellular electron carriers such as NADH and NAD^+^ can be considered a positive approach to enhancing energy generation in bioelectrochemical systems. In this regard, Han et al. regenerated NADH by genetically engineered *Clostridium ljungdahlii*, and increased the maximum power density of the MFC more than twofold ^12^. Moreover, It was demonstrated that the electricity generation of *Synechocystis* depended on NADH production, which is profoundly affected by various metabolic pathways^11^. Therefore, concerning the vital role of metabolic modeling to identify intracellular constraints and switch the cellular metabolism to the generation of NADH, a regulatory defined medium can be developed to shift the cell metabolism to the object of interest^13^.

In this study, a system-oriented strategy based on metabolic modeling is used to design a regulatory defined medium, reducing the intracellular constraints in the bioelectricity generation of *Synechocystis* via the cellular metabolism alteration to improve the NADH generation. Thus, a comprehensive investigation is achieved to identify the key reactions that play a critical role in enhancing electricity production in *Synechocystis* via a linear algorithm for finding multiple optimal solutions (LAMOS)^14^. Consequently, the predicted reactions are regulated by adding enzyme regulators to the culture medium. These compounds regulate the enzyme activity of the target reactions and are found in the BRENDA database^15^. Finally, regulators' effect on BPV performance is evaluated by comparing polarization and power density curves under various conditions.

## Material and methods

### Metabolic model and in silico simulation

A genome-scale metabolic network of *Synechocystis*, named iJN678^16^, was used to investigate the metabolic pathways. The COBRA Toolbox and the GLPK package were applied to MATLAB 2017b to run the metabolic modeling^17^. Optimizing the intracellular electrons generation rates was done by defining the NADH production rates as the objective function. The lower bounds of intracellular reversible and irreversible reactions were applied to -1000 and 0 mmol/gDCW/h, respectively. Moreover, the upper bounds of all intracellular reactions were set to 1000 mmol/gDCW/h. Photoautotrophic conditions were simulated by constraining the photon uptake rate to 52 mmol/gDCW/h, and bicarbonate uptake rate was set to 3.7 mmol/gDCW/h as the only carbon source. The lower and upper bounds of exchange reactions including Co^2+^, Fe^2+^, Fe^3+^, H^+^, Na^+^, Ni^2+^, Cu^2+^, Zn^2+^, Ca^2+^, Mg^2+^, Mn^2+^, O_2_, H_2_O, Molybdate, Potassium, Nitrate, Phosphate, and Sulfate remained unconstrained by -1000 and 1000 mmol/gDCW/h, respectively. Hence the uptake and secretion of the mentioned metabolites were allowed^16^.

### Identifying effective reactions and designing the regulatory defined medium

**Figure 1:**
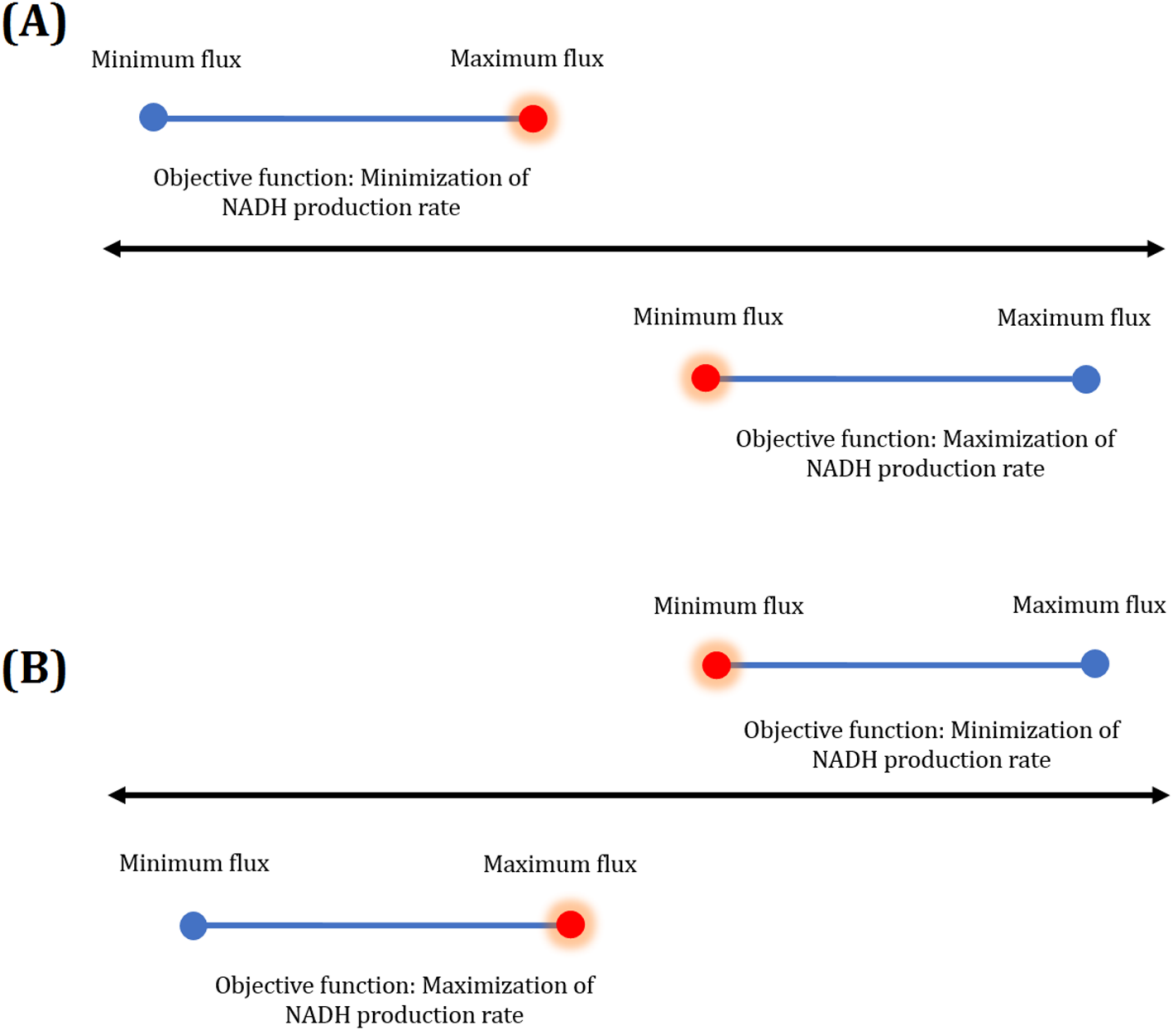
Comparison of flux variability for each reaction at the minimum and maximum rates of NADH production reaction to finding appropriate reactions for (**A**) up-regulation, and (**B**) down-regulation. Compared rates were highlighted in red.

As mentioned earlier^14^, LAMOS^18^ was employed to calculate multiple optimal flux distributions and determine effective reactions for NADH overproduction. Besides, it was previously reported that the biomass concentration of *Synechocystis* in the electrochemical cell should not reduce significantly because it would lead to a low power generation^19^; therefore, reactions that their regulation causes a high reduction of the growth rate are not appropriate candidates for manipulation. According to this point, the optimal biomass formation rate was calculated using FBA^20^ (0.0884 h^−1^), and the growth rate was bound to 90% of the optimal growth rate in the metabolic model to ensure finding bottleneck genes in which manipulating their enzymes for overproduction of NADH does not result in a significant decrease in cell density. Moreover, a threshold of 10^−8^ was considered to determine active reactions. Thus, 10,000 optimal solutions were found for two conditions, including the maximum and the minimum rates of NADH production (i.e., ${\text{NADH}} \to {\text{NAD}}^{ + } + {\text{H}}^{ + } + 2{\text{e}}^{ - }$). Then, for each mentioned condition, the flux variability for each reaction was determined (Fig. 1), and two criteria were applied to identify the candidate reactions for up or down regulations.

As demonstrated in Fig. 1, a reaction is suitable to be up-regulated if its minimum flux in the maximum case (i.e., maximum NADH production rate) is higher than the maximum flux in the minimum case (i.e., minimum NADH production rate). In contrast, a reaction is proper to be down-regulated if its minimum flux in the minimum case is higher than the maximum flux in the maximum one.

The activity difference for each reaction was regarded as the second criterion and was defined as a fraction of entire optimal solutions wherein the reaction was indicated to be active. Therefore, those reactions were taken into consideration that possessed the absolute value of activity difference equal to 1. In this regard, the activity difference of 1 for a reaction indicated that the reaction was entirely active in every 10,000 optimal solutions of maximum NADH production while it was completely inactive in all optimal solutions of minimum NADH production. On the other hand, the reaction, which activated and inactivated entirely in every 10,000 optimal solutions of minimum and maximum NADH production, respectively, possessed the activity difference of -1.

The effect of up (down)-regulation of the predicted reactions was experimentally investigated by adding regulators to the culture medium. These regulatory compounds found in the BRENDA database affected the target enzyme activity controlling the target reaction, which is predicted to result in the overproduction of NADH and, consequently, electricity enhancement. Therefore, the investigation of the regulators' impact on electrons overproduction by *Synechocystis* is another goal of the study.

### Bio-photovoltaic cell (BPV) assembly

**Figure 2:**
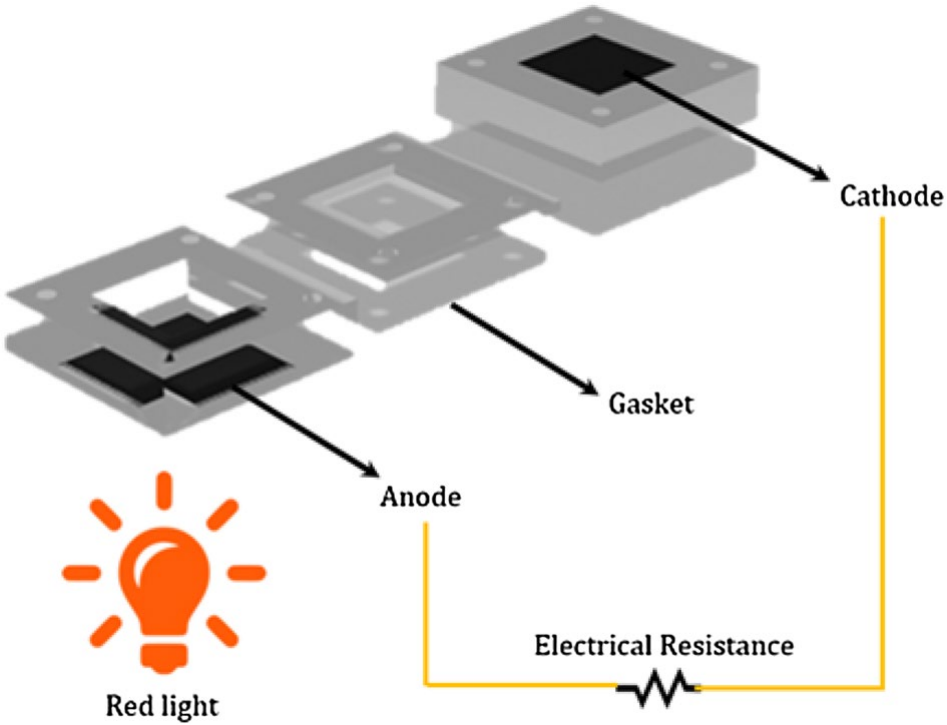
The schematic of the bio-photovoltaic device (BPV).

The air–cathode single chamber BPV with a working volume of 8.8 cm^3^, height of 2.2 cm, and electrodes surface of 4 cm^2^ was fabricated. The anodic and cathodic chambers were fabricated from polymethyl methacrylate (PMMA). The graphite-coated stainless steel mesh (mesh size 400) was used as the anode and cathode electrodes^21^. The cathode was treated by the procedures described in^22^ to attain 0.5 mg cm^−2^ Pt loading. A schematic of the BPV is shown in Fig. 2.

### Microorganism and culture media

*Synechocystis* was obtained from Ariyan Gostar Research Corporation. All cultivation experiments conducted in the BG-11 medium described in the work of Bombelli et al.^7^. In this regard, liquid seed culture was obtained periodically, which was then used for the inoculation of fresh liquid culture. Subsequently, 2 ml of seed culture was inoculated into 100 ml flasks containing 18 ml of fresh BG-11. All incubations were carried out at 150 rpm and 29 °C, which were continuously kept under the illumination of a red light bulb (630 nm) with 6000 lux lighting. It was demonstrated that red light wavelengths have a significant impact on maintaining photosynthetic reactions^23^.

### Inoculation and BPV operation

In all experiments, after five days of cultivation, cyanobacterial cells were harvested during exponential growth ($OD_{750 } = 3.5 \pm 0.1$, Fig. S1) and centrifuged for 15 min at 3000 g. Then, regarding the experiment to be studied, *Synechocystis* cells resuspended in fresh BG-11 or regulatory BG-11. With regard to the regulatory BG-11 medium, the selected regulatory compounds were supplemented to fresh BG-11 according to the concentrations obtained from Brenda to evaluate their effect on electricity generation. Thus, the BPV was inoculated by different culture media types under the batch mode operation and open circuit conditions. The inoculation continued until a stable cell potential peak was obtained, indicating successful microbial enrichment fulfillment and cyanobacterial cells stabilization. The bioelectrochemical experiments were conducted under 6000 lux lighting of red light (630 nm). High-intensity lighting and red light illumination were reported to have a positive effect on increasing power generation of the bioelectrochemical systems^19^.

## Results

### Identifying the effective reactions in NADH production

**Figure 3:**
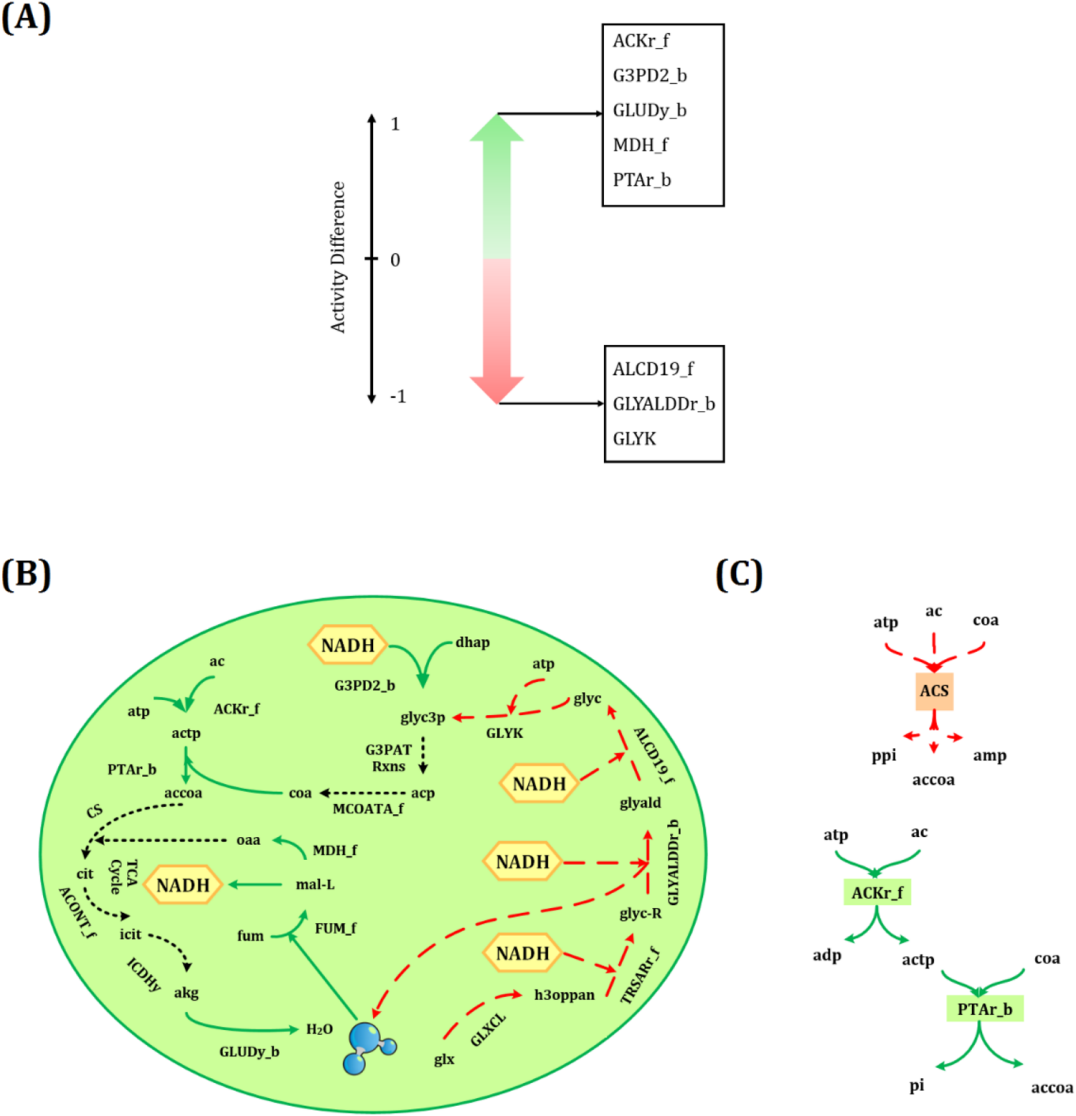
(**A**) Appropriate reactions for up-regulation (top) and down-regulation (bottom) predicted by the proposed systemic approach under phototrophic condition. (**B**) An overview of the metabolic pathways influencing NADH production; the reactions indicated by green arrows are suitable candidates for up-regulation, whereas red dashed lines should be down-regulated. Besides, reactions that do not contribute to NADH enhancement were shown with the black dotted line. (**C**) Acetate consumption via ACS, and series reactions of ACKr_f and PTAr_b. The suffixes _f and _b were used for reactions that occurred in the forward and backward directions, respectively. The entire name of all reactions and metabolites was given in the Supplementary file (Tables S3 and S4).

The appropriate reactions contributing to an increase in the intracellular NADH generation of *Synechocystis* were predicted and shown in Fig. 3A. The reactions depicted on the top possessed the activity difference of 1. Furthermore, their minimum flux under maximum NADH production rate is more than their maximum flux under the minimum NADH production rate (Fig. 1A), and thus there were proposed for up-regulation. In contrast, down-regulation should be considered for those reactions revealed on the bottom with the activity difference of -1 in which their minimum flux under minimum NADH production rate is more than the maximum flux under maximum NADH production rate (Fig. 1B). The maximum and minimum NADH production rates were calculated to be 2.1 and 0 mmol/gDCW/h, respectively. The predicted reactions were prepared in more detail in the Supplementary file (Tables S1 and S2). Moreover, a complete name of all reactions and metabolites were presented in the Supplementary file (Tables S3 and S4), respectively.

The predicted effective metabolic pathways for enhancing NADH are schematically represented in Fig. 3B. Herein, two crucial metabolic pathways for converting acetate (ac) to acetyl-CoA (accoa) that possessed a significant activity difference were selected. As shown in Fig. 3C, acetate could be consumed via the acetyl-CoA synthetase (ACS) by converting ATP to AMP. Besides, this metabolite can also be used through a series of reactions, including acetate kinase (ACKr_f) and phosphotransacetylase (PTAr_b), by conversion of ATP to ADP (Fig. 3C). Thus, acetate kinase and phosphotransacetylase were recommended to produce acetyl-CoA, which then enters the TCA cycle and causes NADH to form via malate dehydrogenase (MDH_f).

The systemic approach also recommended that glycerol 3-phosphate (glyc3p) played a vital role in NADH generation. As demonstrated in Fig. 3B, a red dashed line that starts with glyoxalate carboligase pathway (GLXCL) and ends with glycerol kinase (GLYK), consumed three moles NADH along with one mole of ATP to produce glycerol 3-phosphate. These pathways occur as a series of reactions that each reaction possesses the same flux through the metabolic network leading to 0 mmol/gDCW/h NADH generation (Table 1). Therefore, they are regarded as proper candidates to be down-regulated. Moreover, glycerol 3-phosphate could also be produced by glycerol-3-phosphate dehydrogenase (G3PD2_b), which consumes only one mole of NADH without ATP consumption. Green arrows show this pathway in Fig. 3B. Glycerol-3-phosphate dehydrogenase possesses the same reaction rates as the mentioned series reactions but leads to 2.1 mmol/gDCW/h NADH generation (Table 1). Thus, its up-regulation should be taken to prevent NADH and ATP loss via other metabolic pathways.

**Table 1:** Comparison of reaction rates for generating glycerol 3-phosphate.

Reaction name	Reaction description	Reaction of G3PD2_b and the objective function are maximal (mmol/gDCW/h)	Reaction of G3PD2_b and the objective function are zero (mmol/gDCW/h)	Activity difference
GLXCL	Glyoxalate carboligase	0	0.0138	− 0.9618
TRSARr_f	Tartronate semialdehyde reductase	0	0.0138	− 0.9618
GLYALDDr_b	D-glyceraldehyde dehydrogenase	0	0.0138	− 1
ALCD19_f	Alcohol dehydrogenase	0	0.0138	− 1
GLYK	Glycerol kinase	0	0.0138	− 1
G3PD2_b	Glycerol-3-phosphate dehydrogenase	0.0138	0	1
NADH_pro		2.1	0	1

The suffixes _f and _b indicate the forward and backward directions of a reaction, respectively. The fluxes are presented in the unit of mmol/gDCW/h.

A further investigation into metabolic pathways depicted in (Fig. 3B), showed that the generated glycerol 3-phosphate produces acyl carrier protein (acp), which is then used to form CoA, which can be utilized by (PTAr_b). Furthermore, the hypothetical glycerol-3-phosphate acyltransferase reactions (G3PAT Rxns) comprise nine reactions generating acyl carrier protein, in which their details were mentioned in the Supplementary file (Table S5).

Moreover, the mechanism of extracellular electron transfer (EET) in cyanobacteria has been a subject of study in recent years, leading to conflicting reports^24^.

Gorby et al.^25^ reported the presence of conductive nanowire appendages for *Synechocystis*, which was formed under low CO_2_ conditions. The filaments thickness was reported about 100–150 nm, which was not consistent with individual type IV pili. The type IV pili of *Synechocystis* reported a thickness of 6–8 nm thick^26^. Later on, Thirumurthy et al.^27^ reported that type IV pili were not required for photocurrent generation. McCormick et al.^5^ revealed direct electron transfer of *Synechocystis* by developing a mediatorless BPV system. The direct electron transfer of *Synechocystis* to the anode was also reported by Cereda et al.^9^. However, it was not yet cited which cellular components responsible for transferring electrons to the electrode. Mao et al.^11^ used computational metabolic techniques to study the maximum current outputs of *Synechocystis* in different electron transfer cases. The highest current output production was reported for plastoquinol and c-type cytochrome. Saper et al.^28^ reported that *Synechocystis* could secrete some undefined small molecules to shuttle electrons to the anode. Similarly, Shlosberg et al.^29^ showed that *Synechocystis* employed endogenous NADPH as an electron mediator to generate bioelectricity in bio-photoelectrochemical cells.

According to the mentioned points, the main extracellular electron transfer mechanism of *Synechocystis* has not been recognized yet. To this end, future studies should be carried out to investigate which mechanism mostly governs the extracellular electron transport of *Synechocystis*.

### Microbial enrichment and open circuit potential (OCP) monitoring

**Figure 4:**
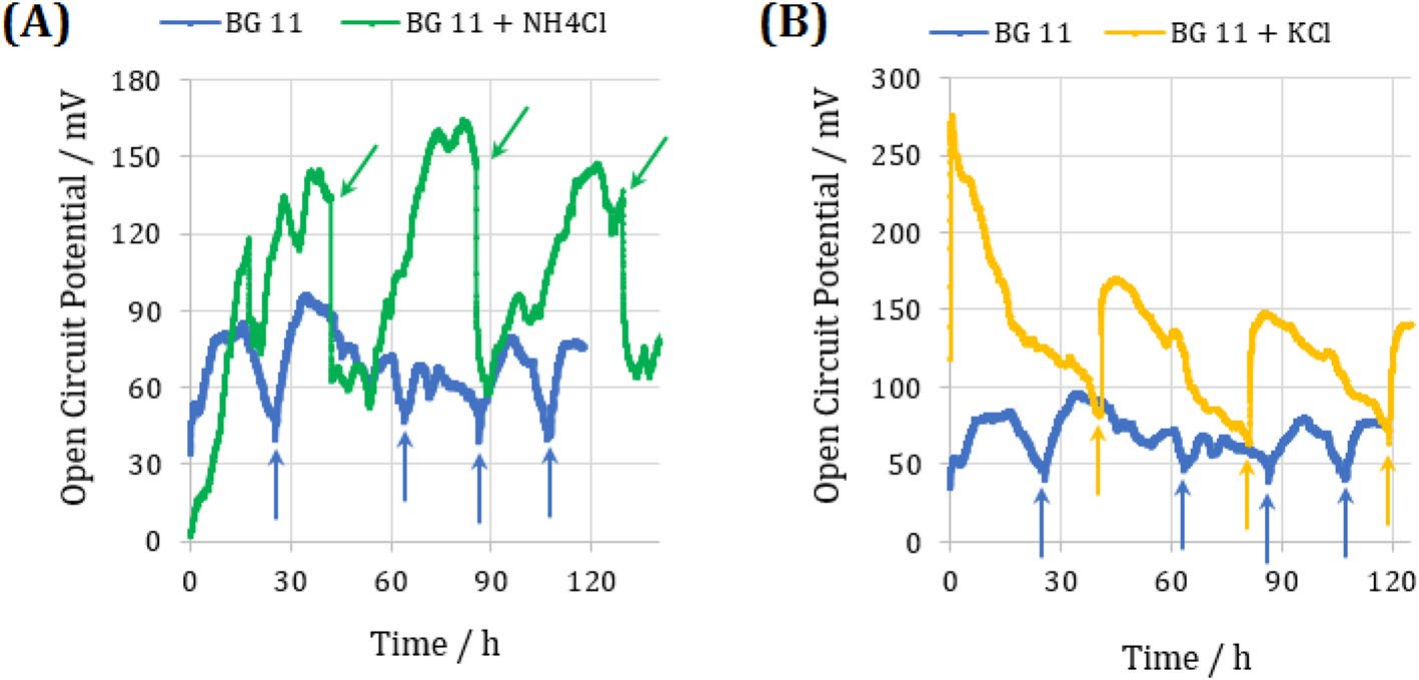
The open circuit potential (OCP) evolution of three used suspensions for microbial enrichment. (**A**) *Synechocystis* + BG-11 and *Synechocystis* + BG-11 + 40 mM NH_4_Cl, (**B**) *Synechocystis* + BG-11 and *Synechocystis* + BG-11 + 50 mM KCl.

With regard to the introduced reactions by metabolic modeling in Fig. 3A, phosphotransacetylase and glutamate dehydrogenase were taken into consideration. As reported in Brenda and previously published studies, 40 mM NH_4_Cl resulted in threefold stimulation of phosphotransacetylase^30^, and 50 mM KCl led to an increase in 170% activity of glutamate dehydrogenase^31^. Hence, three suspensions for microbial enrichment were used including *Synechocystis* + BG-11, *Synechocystis* + BG-11 + 50 mM KCl, and *Synechocystis* + BG-11 + 40 mM NH_4_Cl. Having concluded an effective biofilm formation during open circuit conditions^32^, which established a uniform biofilm facilitating substrate diffusion and electron transfer, the microbial enrichment of the BPV has been done by monitoring the open circuit potential (OCP). Thus, the OCP evolution of the three culture media was shown in Fig. 4.

Considering the OCP of the sole culture medium of BG-11, the addition of activators (i.e., KCl or NH_4_Cl) bring about a significant increase in the OCP of the BPV. Since Nernst's equation depicting the effective parameters on the electrochemical OCP, the higher cell potentials strongly depend on the activation of redox species implying the critical role of KCl and NH_4_Cl in this increment.

By observing a noticeable decrease in OCP, the fresh medium was injected into the BPV, as shown by arrows in Fig. 4. The replacement of the fresh culture medium with the old one causes an increase in the OCP trend. Since the BPV operated in the batch mode with high cell density ($OD_{750 } = 3.5 \pm 0.1$), the potential reduction can be attributed to the depletion of nutrients; thus, fresh medium injection compensates for the potential drop. This phenomenon was also shown previously in the work of Madiraju et al.^19^, in which replenishing minerals led to an increase in power density. Moreover, for the culture media with activator, this abrupt growth in the OCP was higher than the sole culture medium of BG-11. A comparison between Fig. 4A, B reveal that the culture medium of *Synechocystis* + BG-11 + 40 mM NH_4_Cl obtained the higher OCP and more prolonged stationary phase. This issue puts the emphasis on the more impact of this culture medium on the cell potential compared to the BG-11 + 50 mM KCl.

### Polarization and power density curves

**Figure 5:**
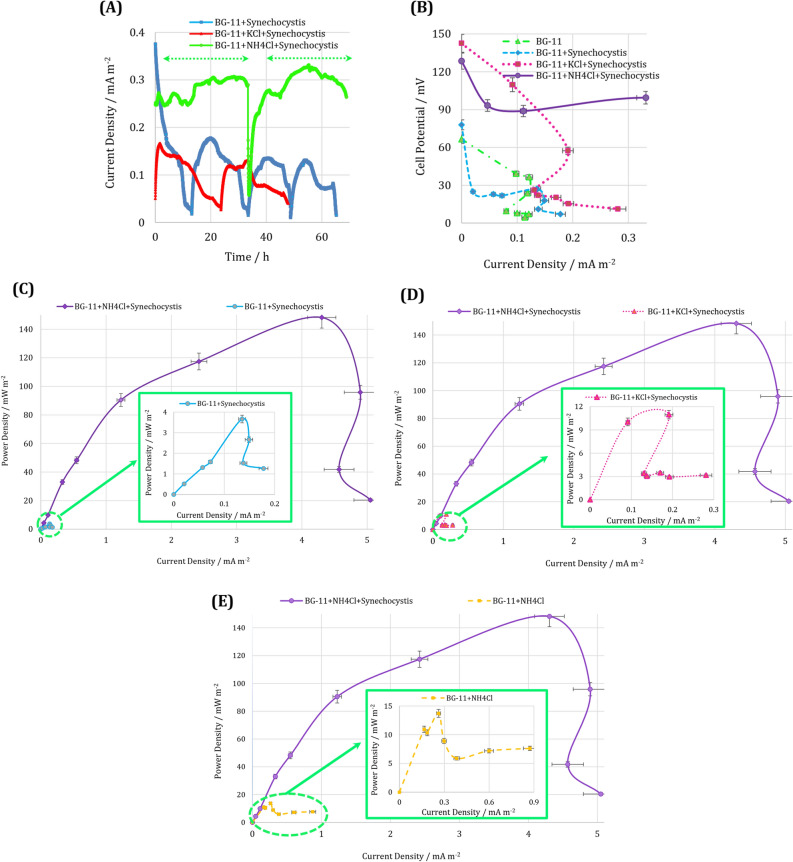
(**A**) The current evolution, (**B**) polarization and (**C**), (**D**), and (**E**) power density curves for different culture media. The error bars represent the variation of obtained results among repeated experiments.

The effect of external resistance on the BPV performance fed with various culture media types was investigated by monitoring current evolution. Figure 5A depicted the current evolution of *Synechocystis* for BG-11, BG-11 + KCl, and BG-11 + NH_4_Cl culture media at 500, 500, and 750 kΩ external resistances, respectively. The presence of KCl increased the maximum produced current of the BPV by more than 8.8% compared with its value when the initial culture medium of BG-11 was fed. The addition of NH_4_Cl to BG-11 had a remarkable influence on the current production and increased the maximum current density of the cell more than twofold compared with the BPV fed by BG-11. This incremental phenomenon was obtained at a higher external resistance of 750 kΩ, which was comparable to the other media implying more increase have to be obtained in the same external resistance (i.e., 500 kΩ).

Additionally, the stationary phase in the current evolution (shown by two dashed horizontal arrows in Fig. 5A) of the BG-11 + NH_4_Cl fed BPV was longer compared to the BPV cultured BG-11 and BG-11 + KCl, indicating a stable current density by extension feed replacement.

The BPV overpotentials (including activation, ohmic, and concentration) can be investigated by applying different external resistances to polarize the cell. The polarization curves of four types of BPV feed was illustrated in Fig. 5B. The abrupt reductions of cell potentials in the polarization curves of BG-11 + *Synechocystis* and BG-11 + KCl + *Synechocystis* indicate the high activation overpotential to extract the electron from the cellular metabolism. This type of overpotential was remarkably higher than two other overpotentials (i.e., ohmic and concentration overpotentials) characterized at the middle and end of polarization curves. Activation overpotential represents the energy that microbes required to transfer electrons from their surface to the electrode^33^. These high drops corroborate the hypothesis presented in previous researches^5,9^, indicating the incapability of *Synechocystis* to transfer electrons from its membrane due to the competition of numerous metabolic pathways for energy resources. Moreover, the absence and presence of cyanobacteria in the BG-11 culture medium demonstrated the incapability of *Synechocystis* in generating electrons. Although the addition of KCl increased the current density, the initial sharp reduction could not be compensated. However, for the BPV fed BG-11 + NH_4_Cl + *Synechocystis*, the cell potential reduction was exceedingly made up, revealing the impressive role of NH_4_Cl to compensate for the activation loss of metabolic reactions and extract electrons from light and water.

Furthermore, the higher current densities obtained at lower external resistances show the augmented electron production and accentuate the stimulation of cell electrogenesis metabolic pathways. Low activation losses owned to the fact that phosphotransacetylase had a crucial role in converting acetate to acetyl-CoA, assisting the cell to maintain a balance between biosynthesis and energy production^34^. Therefore, the overexpression of this enzyme by NH_4_Cl could facilitate electrons transfer.

Moreover, the occurrence of the overshoot phenomenon resulting from sudden electron depletion at low external resistances that cannot be compensated by microorganisms^35^ was observed in the BPV fed with all four types of culture media (Fig. 5B). The overshoot in the BPV fed with BG-11 + KCl + *Synechocystis* led to a more than 31% decrease in the current density (from 0.1913 to 0.1306 mA m^−2^). The culture medium used NH_4_Cl as an activator showed a better performance in compensating electron depletions and only brought about a 6.6% decrease in the produced current. This is another evidence emphasizing the crucial role of NH_4_Cl, which improves *Synechocystis* electrogenesis metabolic pathways.

The power density curves for three culture media were shown in Fig. 5C–E. The BPV fed by BG-11 + NH_4_Cl + *Synechocystis* achieved the maximum power density of 148.27 mW m^−2^, which is more than 40.5-fold of what was obtained for the BPV fed with BG-11 + *Synechocystis* (Fig. 5C).

The maximum produced power density for BG-11 + KCl + *Synechocystis* was 10.97 mW m^−2^ (Fig. 5D). Despite the higher salt concentration, this power density was 13.5 times lower than the BPV fed by BG-11 + NH_4_Cl + *Synechocystis*. Achieving a higher power density by NH_4_Cl compared to KCl can be related to the fact that KCl had an adverse effect on some critical metabolic pathways, which is discussed in the Supplementary file (Table S6). Therefore, with regard to the role of KCl in the stimulation of glutamate dehydrogenase, it cannot be helpful in electrogenesis metabolism as much as NH_4_Cl.

Furthermore, to scrutinize the effect of NH_4_Cl on diminishing intracellular constraints, *Synechocystis* was removed from the BPV. In this case, the maximum produced power density for BG-11 + NH_4_Cl was 13.72 mW m^−2^ (Fig. 5E). This power density was more than tenfold lower than the maximum power density of the BPV fed with BG-11 + NH_4_Cl + *Synechocystis*. The abiotically produced power density is mainly attributed to the medium salinity^33,36^. Therefore, the presence of NH_4_Cl results in the metabolic stimulation of *Synechocystis* rather than the anolyte conductivity improvement. Thus, the addition of NH_4_Cl can play a critical role in shifting the *Synechocystis* metabolism to produce more electricity.

Finally, the average operating time of *Synechocystis* for each external resistance was about 70 h for BG-11 + NH_4_Cl, 52 h for BG-11 + KCl, and 40 h for BG-11. The polarization curves of *Synechocystis* for BG-11 + NH_4_Cl was obtained over 36 days compared to approximately 20 days for BG-11 + KCl and over 16 days for BG-11. Thus, the overall period of BPV operation under different culture media was also increased by the addition of regulators.

### Biofilm morphology

**Figure 6:**
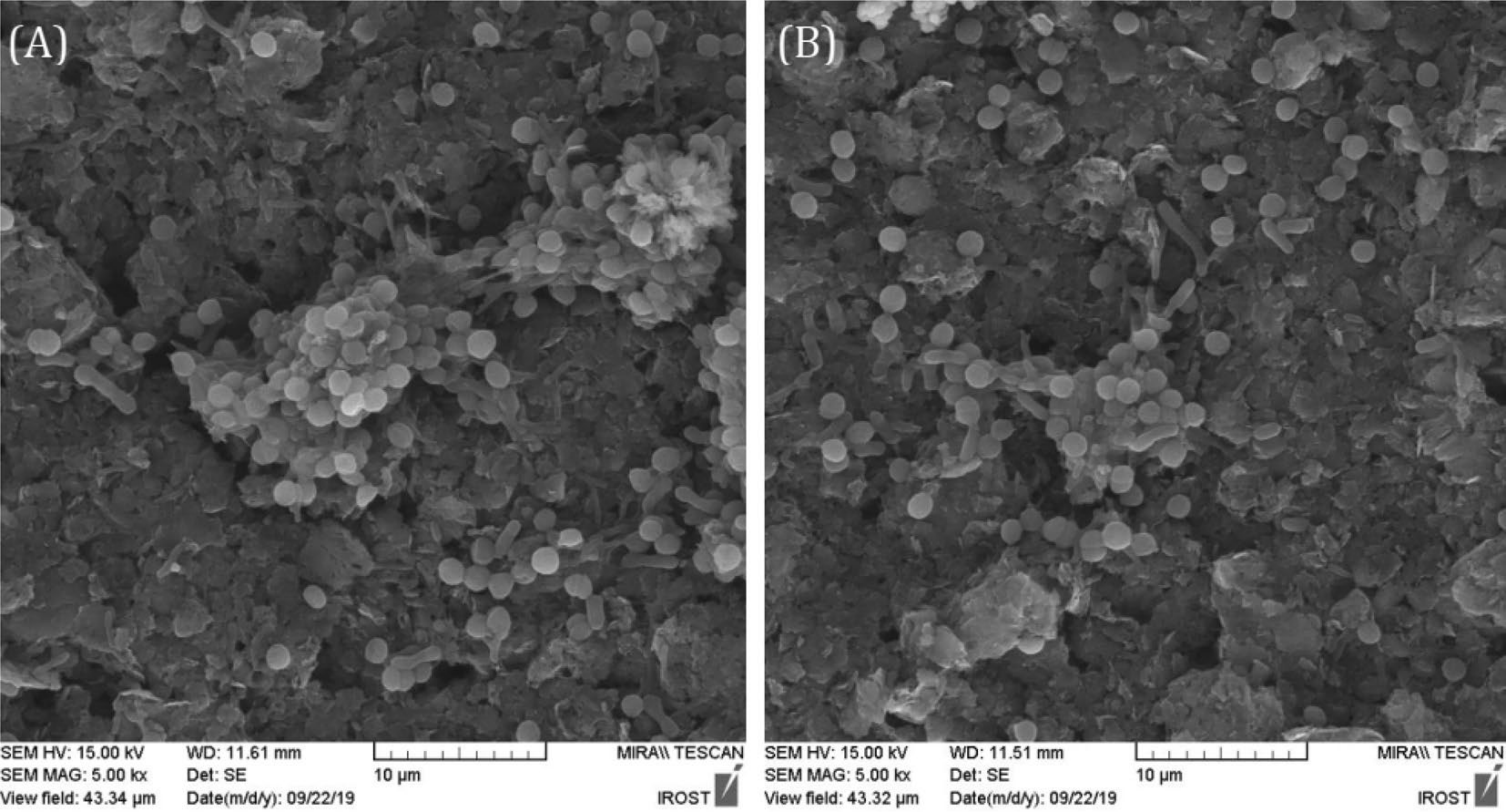
Field emission scanning electron microscopy (FESEM) of *Synechocystis* biofilm in (**A**) BG-11, and (**B**) BG-11 + NH_4_Cl on the anode surface at 5000 magnification.

FESEM micrographs of *Synechocystis* in BG-11 and BG-11 + NH_4_Cl were illustrated in Fig. 6. In both cases, a small portion of cells attached to the anode's surface. This low biofilm formation reported here is similar to those investigated by^5^ in which 8.5% of *Synechocystis* attached to the anode's surface made of indium tin oxide-coated polyethylene terephthalate (ITO-PET). Consequently, the addition of NH_4_Cl to BG-11 had a small impact on the biofilm morphology, and the observed power density enhancement is mainly attributed to the improvement of electrogenesis metabolic pathways.

## Discussion

**Figure 7:**
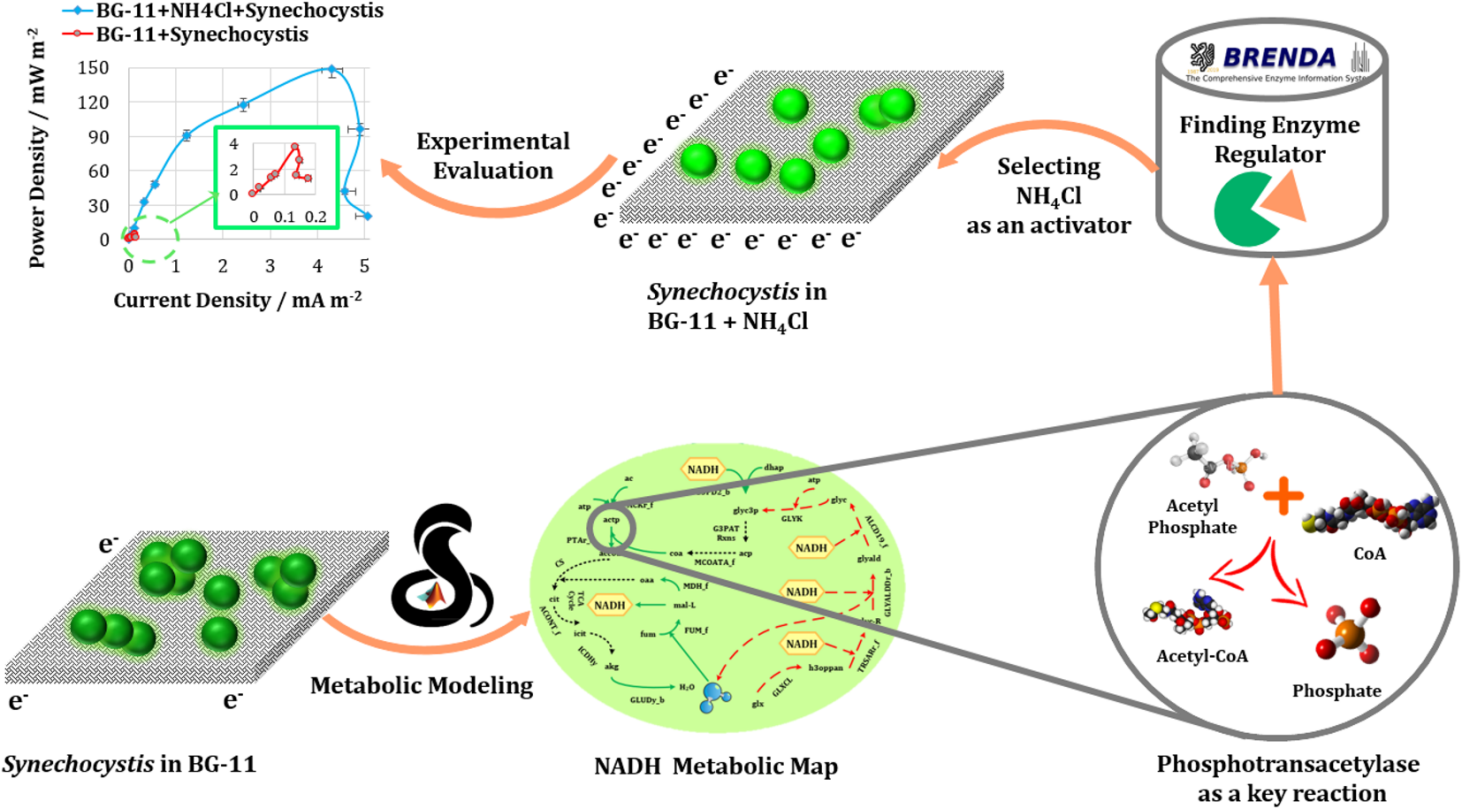
Graphical abstract.

The proposed systemic approach based on metabolic modeling identified bottleneck genes in metabolism for enhancing bioelectricity generation. To this end, LAMOS and BRENDA were applied for the initial screening of existing compounds for switching metabolism to improve NADH production, and therefore increase power density generation (Fig. 7). It is important to note that proposed system-oriented strategy is a screening that seeks to make suggestions from a large number of available components which the effect of their addition to the medium can be evaluated. This strategy suggested the components affect the enzyme of key bottleneck genes, and thus, increasing the chance of improving bioelectricity production. In this regard, LAMOS identified the reactions that had the highest potential in improving bioelectricity production, listed in Tables S1 and S2. Then, for each reaction, the potential regulators in the BRENDA database were searched. Besides, in addition to the experimentally reported effect of the regulator on the bottleneck genes, the regulator may be effective on some other enzymes; therefore, assessing the effect on the final product, which is the production of electrons, maybe the best solution. This method has also been implemented successfully in previous studies^13,37^ leading to improved production.

After an in-depth screening of all components in the BRENDA database, phosphotransacetylase, and glutamate dehydrogenase were selected to be experimentally investigated due to the following reasons. Firstly, since it is undeniable that regulators can affect other enzymes' activity as well^13^; thus, the adverse effect of regulators on other metabolic pathways in improving power generation was investigated. In other words, as it was shown in Table S6, ammonium chloride, which is an activator of phosphotransacetylase, did not have a substantial adverse effect on other critical metabolic pathways. However, potassium chloride, an activator of glutamate dehydrogenase, possessed an adverse effect on some critical metabolic pathways, including the inhibitory effect on oxidative phosphorylation pathways (Table S6). It was concluded that using the regulators that had an adverse effect on crucial pathways could decrease the bioelectricity production, albeit its positive effect on the targeted reaction. It is worth mentioning that the detailed effects of additives on cellular metabolism cannot be accurately evaluated, but with the aid of BRENDA, an attempt has been made to provide an estimate of the regulator’s effect on cellular metabolism, and components screening has been carried out based on this. Furthermore, for some enzymes, such as glycerol-3-phosphatedehydrogenase, no regulators were specified in the BRENDA database. Most recently, Lai et al.^24^ also developed a recipe for BG11 (nBG11), compatible with the BPV operation. The nBG11 medium was modified by removing organic pH buffers (HEPES/TES), optimizing MnCl_2_ concentration, and chelating trace metal mix solution (TMM) with EDTA. Optimizing culture medium using 1 mM ferricyanide as the mediator led to the long‐term photo‐current generation of about 20 µA from *Synechocystis* during repeated light–dark cycles for over 12 days. Similarly, in our study, redesigning the culture medium, particularly the supplementation of NH_4_Cl, generated a longer stationary phase (Fig. 4A), which shows a more stable current density. Moreover, Bombelli et al.^38^ were also supplemented BG-11 with NaCl to study bioelectricity generation of *Synechocystis*; therefore, the closest minerals to NaCl were obtained and studied.

Our results demonstrate that adding activators could provoke the *Synechocystis* as a strain engaged in noticeable intrinsic metabolic losses to reduce its activation overpotential significantly and accelerate bioelectricity generation. The overexpression of the phosphotransacetylase by NH_4_Cl exceedingly facilitates electrons transfer and stimulates cell electrogenesis metabolic pathways, which brings about lower activation losses. Although KCl led to an increase in glutamate dehydrogenase activity, the experimental results showed that it could not be helpful in electrogenesis metabolism as much as NH_4_Cl. The investigation of overshoot occurrence in the BPV also demonstrated the culture medium used NH_4_Cl as an activator produced a better performance in compensating electron depletions. Furthermore, the addition of NH_4_Cl had a small effect on the biofilm morphology.

Interestingly, our experimental studies showed that the addition of 40 mM NH_4_Cl resulted in a 49% decrease in optical density (OD750) in the strain culture, which is consistent with the previous study, representing a photodamage^39^. On the other hand, it was reported that ammonium chloride saves up to 30% of the reducing equivalents produced by the photosynthetic light reactions in cyanobacteria^40^. More specifically, nitrate needs to be reduced to ammonium via two sequential reactions catalyzed by nitrate reductase and nitrite reductase to be assimilated by cyanobacteria. The reductions of nitrate to nitrite to ammonium are Fd-dependent, consuming two and eight electrons (Table S7), respectively^41^. Thus, although ammonium chloride was reported to cause photodamage and reducing cell density, its role in reducing intracellular constraints, saving reducing equivalents, and facilitating electrons transfer is undeniable.

Additionally, this study focused on reducing intracellular constraints to enhance bioelectricity generation. However, some researches were conducted to minimize extracellular constraints to improve the BPV performance. Wenzel et al.^42^ enhanced current generation from photosynthetic biofilms of *Nostoc punctiforme* and *Synechocystis* by using porous translucent electrodes in which a twofold increase in current generation was observed. Bombelli et al.^38^ harnessed a high power density of 105 mW m^−2^ for *Synechocystis* by miniaturizing geometries using microfluidic BPV. In microfluidic devices, due to the reduction of the distance between the electrodes, ohmic losses are reduced^33^, and achieving the maximum power density is more feasible. Thus, diminishing intracellular constraints by the method presented in this study together with optimizing the BPV to reduce extracellular constraints, would be a big step towards the industrialization of this technology.

Future research should also aim to improve the electrogenesis metabolic pathways by implementing systems metabolic engineering approach^43^. Zhu et al.^44^ developed a BPV with a constrained electron flow based on a d-lactate mediated consortium consisting of engineered *Synechococcus elongatus* UTEX 2973 and *Shewanella oneidensis* MR-1. The cyanobacteria harvested solar energy to produce d-lactate, which is subsequently used by the exoelectrogenic microorganism, releases electrons from it, and transfers them to the anode for bioelectricity generation. A power density of 150 mW·m^−2^ in a temporal separation setup was produced by implementing this method. Thus, unlike the addition of mediators such as potassium ferricyanide, which is unsuitable for large-scale^7^, it is more interesting to implement the systemic approach to introduce novel microbial interactions capable of sustainable bioelectricity production.

Besides, instead of supplementing the classic inhibitors such as DCMU and DBMIB, which were used to investigate the source of electrons in the photosynthetic organism, the addition of regulators identified by the systemic approach would bring about more benefits by improving electrical power generation. Finally, it is worth to mention that the produced power density of *Synechocystis* is still relatively lower than exoelectrogenic bacteria such as *Shewanella* or *Geobacter*. However, it would be interesting to implement this method to enhancing exoelectrogenic bacteria bioelectricity generation.

## Supplementary Information

Supplementary Information 1.

## Data Availability

All data generated or analysed during this study are included in this published article.
