# Supplementary material for: A system-oriented strategy to enhance electron production of Synechocystis sp. PCC6803 in bio-photovoltaic devices: experimental and modeling insights
Source: Sci Rep. 2021 Jun 10;11:12294. doi: 10.1038/s41598-021-91906-9 (PMC8192556; doi:10.1038/s41598-021-91906-9)
Supplement: Supplementary file 1 — Supplementary Information 1. [file 41598_2021_91906_MOESM1_ESM.docx]

**A system-oriented strategy to enhance electron production of *Synechocystis* sp. PCC6803 in bio-photovoltaic devices: experimental and modeling insights**

Hossein Firoozabadi ^1^, Mohammad Mahdi Mardanpour ^2^, Ehsan Motamedian ^1,*^

^1^ Department of Biotechnology, Faculty of Chemical Engineering, Tarbiat Modares University, P.O. Box 14115-143, Tehran, Iran.

^2^ Department of chemical and petroleum engineering, Sharif University of Technology, Tehran, Iran

**Table S1.** Predicted reactions for up-regulation to improve NADH production. The fluxes are presented in the unit of mmol/gDCW/h.

| Reaction Name | Reaction Description | Formula | Sub-system | Gene | EC Number | Activity Difference | Maximized NADH production | | Minimized NADH production | |
| --- | --- | --- | --- | --- | --- | --- | --- | --- | --- | --- |
|  |  |  |  |  |  |  | Maximum flux | Minimum flux | Maximum flux | Minimum flux |
| ACKr_f | Acetate kinase | atp[c] + ac[c] <=> adp[c] + actp[c] | Pyruvate metabolism | *sll1299* | 6.2.1.1 | 1 | 0.0155 | 0.0155 | 0 | 0 |
| G3PD2_b | Glycerol-3-phosphate dehydrogenase | h[c] + nadh[c] + dhap[c] <=> nad[c] + glyc3p[c] | Glycerolipid metabolism | *slr1755* | 1.1.1.94 | 1 | 0.0138 | 0.0138 | 0 | 0 |
| GLUDy_b | Glutamate dehydrogenase | h[c] + nadph[c] + akg[c] + nh4[c] <=> h2o[c] + nadp[c] + glu-L[c] | Nitrogen metabolism | *slr0710* | 1.4.1.4 | 1 | 0.4534 | 0.4534 | 0 | 0 |
| MDH_f | Malate dehydrogenase | nad[c] + mal-L[c] <=> h[c] + nadh[c] + oaa[c] | Citrate cycle (TCA cycle) | *sll0891* | 1.1.1.37 | 1 | 0.0674 | 0.0674 | 0 | 0 |
| PTAr_b | Phosphotransacetylase | coa[c] + actp[c] <=> accoa[c] + pi[c] | Pyruvate metabolism | *slr2132* | 2.3.1.8 | 1 | 0.0155 | 0.0155 | 0 | 0 |

The suffixes _f and _b indicate the forward and backward directions of a reaction, respectively.

**Table S2.** Predicted reactions for down-regulation to improve NADH production. The fluxes are presented in the unit of mmol/gDCW/h.

| Reaction Name | Reaction Description | Formula | Sub-system | Gene | EC Number | Activity Difference | Maximized NADH production | | Minimized NADH production | |
| --- | --- | --- | --- | --- | --- | --- | --- | --- | --- | --- |
|  |  |  |  |  |  |  | Maximum flux | Minimum flux | Maximum flux | Minimum flux |
| ALCD19_f | Alcohol dehydrogenase | h[c] + nadh[c] + glyald[c] <=> nad[c] + glyc[c] | Glycerolipid metabolism | *sll0990* | 1.1.1.1 | -1 | 0 | 0 | 0.2128 | 0.0138 |
| GLYALDDr_b | D-glyceraldehyde dehydrogenase | 2 h[c] + nadh[c] + glyc-R[c] <=> nad[c] + h2o[c] + glyald[c] | Glycerolipid metabolism | *slr0091* | 1.2.1.3 | -1 | 0 | 0 | 0.2128 | 0.0138 |
| GLYK | Glycerol kinase | atp[c] + glyc[c] -> adp[c] + h[c] + glyc3p[c] | Glycerolipid metabolism | *slr1672* | 2.7.1.30 | -1 | 0 | 0 | 0.2128 | 0.0138 |

The suffixes _f and _b indicate the forward and backward directions of a reaction, respectively.

**Table S3.** Full details of the reactions presented in Fig. 3.

| Reaction Abbreviation | Full Reaction Name | Formula | Activity Difference | Metabolic model suggestion for NADH production |
| --- | --- | --- | --- | --- |
| ACKr_f | Acetate kinase | atp[c] + ac[c] <=> adp[c] + actp[c] | 1 | To be up-regulated. |
| PTAr_b | Phosphotransacetylase | coa[c] + actp[c] <=> accoa[c] + pi[c] | 1 | To be up-regulated. |
| MDH_f | Malate dehydrogenase | nad[c] + mal-L[c] <=> h[c] + nadh[c] + oaa[c] | 1 | To be up-regulated. |
| GLUDy_b | Glutamate dehydrogenase | h[c] + nadph[c] + akg[c] + nh4[c] <=> h2o[c] + nadp[c] + glu-L[c] | 1 | To be up-regulated. |
| G3PD2_b | Glycerol-3-phosphate dehydrogenase | h[c] + nadh[c] + dhap[c] <=> nad[c] + glyc3p[c] | 1 | To be up-regulated. |
| FUM_f | Fumarase | h2o[c] + fum[c] <=> mal-L[c] | 0.0068 | To be up-regulated. |
| ALCD19_f | Alcohol dehydrogenase | h[c] + nadh[c] + glyald[c] <=> nad[c] + glyc[c] | -1 | To be down-regulated. |
| GLYALDDr_b | D-glyceraldehyde dehydrogenase | 2 h[c] + nadh[c] + glyc-R[c] <=> nad[c] + h2o[c] + glyald[c] | -1 | To be down-regulated. |
| GLYK | Glycerol kinase | atp[c] + glyc[c] -> adp[c] + h[c] + glyc3p[c] | -1 | To be down-regulated. |
| GLXCL | Glyoxalate carboligase | h[c] + 2 glx[c] -> co2[c] + 2h3oppan[c] | -0.9618 | To be down-regulated. |
| TRSARr_f | Tartronate semialdehyde reductase | h[c] + nadh[c] + 2h3oppan[c] <=> nad[c] + glyc-R[c] | -0.9618 | To be down-regulated. |
| ACS | Acetyl-CoA synthetase | atp[c] + coa[c] + ac[c] -> ppi[c] + accoa[c] + amp[c] | -0.9637 | To be down-regulated. |
| MCOATA_f | Malonyl-CoA-ACP transacylase | ACP[c] + malcoa[c] <=> malACP[c] + coa[c] | 0 | Neutral |
| CS | Citrate synthase | h2o[c] + accoa[c] + oaa[c] -> h[c] + coa[c] + cit[c] | 0 | Neutral |
| ACONT_f | Aconitase | cit[c] <=> icit[c] | 0 | Neutral |
| ICDHy | Isocitrate dehydrogenase | nadp[c] + icit[c] -> co2[c] + nadph[c] + akg[c] | 0 | Neutral |

The suffixes _f and _b indicate the forward and backward directions of a reaction, respectively.

**Table S4.** Full details of the metabolites presented in Fig. 3.

| Metabolite Abbreviation | Full Metabolite Name | Metabolite Abbreviation | Full Metabolite Name |
| --- | --- | --- | --- |
| atp[c] | ATP | nadp[c] | Nicotinamide adenine dinucleotide phosphate |
| ac[c] | Acetate | glu-L[c] | L-Glutamate |
| adp[c] | ADP | dhap[c] | Dihydroxyacetone phosphate |
| actp[c] | Acetyl phosphate | glyc3p[c] | Glycerol 3-phosphate |
| coa[c] | Coenzyme A | fum[c] | Fumarate |
| accoa[c] | Acetyl-CoA | glyald[c] | D-Glyceraldehyde |
| pi[c] | Phosphate | glyc[c] | Glycerol |
| nad[c] | Nicotinamide adenine dinucleotide | glyc-R[c] | (R)-Glycerate |
| mal-L[c] | L-Malate | glx[c] | Glyoxylate |
| h[c] | H+ | co2[c] | CO_2_ |
| nadh[c] | Nicotinamide adenine dinucleotide – reduced | h3oppan[c] | 2-Hydroxy-3-oxopropanoate |
| oaa[c] | Oxaloacetate | ppi[c] | Diphosphate |
| nadph[c] | Nicotinamide adenine dinucleotide phosphate – reduced | amp[c] | AMP |
| akg[c] | 2-Oxoglutarate | ACP[c] | acyl carrier protein |
| nh4[c] | Ammonium | malcoa[c] | Malonyl-CoA |
| h2o[c] | H_2_O | malACP[c] | Malonyl-[acyl-carrier protein] |
| cit[c] | Citrate | icit[c] | Isocitrate |

[c] refers to the metabolites located in the cytosol.

**Table S5.** Hypothetical G3PAT Rxns comprising nine reactions generating acyl carrier protein. The fluxes are presented in the unit of mmol/gDCW/h.

| Reaction  Name | Reaction  Description | Formula | Reaction Rates | Activity  Difference |
| --- | --- | --- | --- | --- |
| G3PAT160 | Glycerol-3-phosphate acyltransferase (C16 0) | palmACP[c] + glyc3p[c] -> ACP[c] + 1hdecg3p[c] | 0.0066 | 0 |
| G3PAT161 | Glycerol -3-phosphate acyltransferase (C16 1) | hdeACP[c] + glyc3p[c] -> ACP[c] + 1hdec9eg3p[c] | 0.0005 | 0 |
| G3PAT180 | Glycerol -3-phosphate acyltransferase (C18 0) | ocdcaACP[c] + glyc3p[c] -> ACP[c] + 1odecg3p[c] | 0.0001 | 0 |
| G3PAT181 | Glycerol -3-phosphate acyltransferase (C18 1) | octeACP[c] + glyc3p[c] -> ACP[c] + 1odec11eg3p[c] | 0.0004 | 0 |
| G3PAT181(9) | Glycerol -3-phosphate acyltransferase (C18 1) | octe(9)ACP[c] + glyc3p[c] -> ACP[c] + 1odec9eg3p[c] | 0.0004 | 0 |
| G3PAT182(9_12) | Glycerol -3-phosphate acyltransferase (C18 2) | octe(9_12)ACP[c] + glyc3p[c] -> ACP[c] + 1odec912eg3p[c] | 0.0023 | 0 |
| G3PAT183(6_9_12) | Glycerol -3-phosphate acyltransferase (C18 3) | octe(6_9_12_15)ACP[c] + glyc3p[c] -> ACP[c] + 1odec691215eg3p[c] | 0.0001 | 0 |
| G3PAT183(9_12_15) | Glycerol -3-phosphate acyltransferase (C18 3) | octe(9_12_15)ACP[c] + glyc3p[c] -> ACP[c] + 1odec91215eg3p[c] | 0.0001 | 0 |
| G3PAT184(6_9_12_15) | Glycerol -3-phosphate acyltransferase (C18 4) | octe(6_9_12)ACP[c] + glyc3p[c] -> ACP[c] + 1odec6912eg3p[c] | 0.0021 | 0 |

**Table S6.** Comparison of the potassium chloride and ammonium chloride effect on crucial metabolic pathways of NADH production. The fluxes are presented in the unit of mmol/gDCW/h.

| Reaction Abbreviation | Full Reaction Name | Activity  Difference | The nature of the reaction | Compound Effect | Reference |
| --- | --- | --- | --- | --- | --- |
| CYO1b_syn | Cytochrome c oxidase | 0.9547 | To be activated | The addition of 20 and 50 mM potassium chloride had a 50% inhibitory effect. | ^1-3^ |
| CYO1bpp_syn | Cytochrome c oxidase | 0.7904 | To be activated |  |  |
| GLUDy_b | Glutamate dehydrogenase | 1 | To be activated | The addition of 100 mM potassium chloride had a 45% inhibitory effect. | ^4^ |
| G3PD2_b | Glycerol-3-phosphate dehydrogenase | 1 | To be activated | The addition of 100 mM potassium chloride and 100 mM ammonium chloride had 47% and 37% inhibitory effects, respectively. | ^5^ |
| GLYALDDr_b | D-glyceraldehyde dehydrogenase | -1 | To be inhibited | The addition of 250 mM potassium chloride had the maximum activation effect. | ^6^ |
| PDHcr_f | Pyruvate dehydrogenase | -0.758 | To be inhibited | 4°C: activates the enzyme at concentrations below 1 M of potassium chloride. | ^7^ |

The suffixes _f and _b indicate the forward and backward directions of a reaction, respectively.

50mM potassium chloride led to an increase in 170% activity of glutamate dehydrogenase (NADP^+^)^8^, but as shown in Table S7, it also had an adverse effect on some critical metabolic pathways. In other words, in some pathways that the aim is to increase the activity, potassium chloride had an inhibitory effect and vice versa. However, ammonium chloride had only one reverse effect on the metabolic pathway. Therefore, it can be concluded that potassium chloride had a dual role in increasing and decreasing NADH generation; thus, it cannot improve the BPV performance like ammonium chloride.

**Table S7.** Full details of the reactions converted nitrate to ammonium.

| Reaction Abbreviation | Full Reaction Name | Formula | Consumed electrons |
| --- | --- | --- | --- |
| NAR_syn | Ferredoxin-nitrate reductase | 2 h[c] + 2 fdxr-2_2[c] + no3[c] -> h2o[c] + 2 fdxo-2_2[c] + no2[c] | 2 |
| NOR_syn | Ferredoxin-Nitrite Reductase | 8 h[c] + 6 fdxr-2_2[c] + no2[c] -> 2 h2o[c] + nh4[c] + 6 fdxo-2_2[c] | 6 |


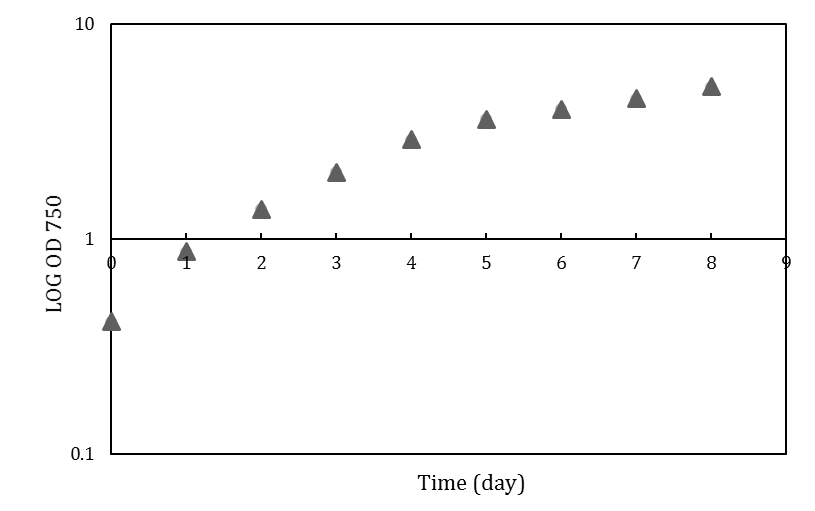


**Figure S1.** The growth curve of *Synechocystis* under photoautotrophic conditions.

**References**

1 DE VRIJ, W., AZZI, A. & KONINGS, W. N. Structural and functional properties of cytochrome c oxidase from Bacillus subtilis W23. *European journal of biochemistry* **131**, 97-103 (1983).

2 Maeshima, M. & Asahi, T. Purification and characterization of sweet potato cytochrome c oxidase. *Archives of biochemistry and biophysics* **187**, 423-430 (1978).

3 Maeshima, M., Hattori, T. & Asahi, T. in *Methods in Enzymology* Vol. 148 491-501 (Elsevier, 1987).

4 Wakamatsu, T., Higashi, C., Ohmori, T., Doi, K. & Ohshima, T. Biochemical characterization of two glutamate dehydrogenases with different cofactor specificities from a hyperthermophilic archaeon Pyrobaculum calidifontis. *Extremophiles* **17**, 379-389 (2013).

5 Kito, M. & Pizer, L. I. Purification and regulatory properties of the biosynthetic L-glycerol 3-phosphate dehydrogenase from Escherichia coli. *Journal of Biological Chemistry* **244**, 3316-3323 (1969).

6 Cao, Y., Liao, L., Xu, X.-w., Oren, A. & Wu, M. Aldehyde dehydrogenase of the haloalkaliphilic archaeon Natronomonas pharaonis and its function in ethanol metabolism. *Extremophiles* **12**, 849 (2008).

7 Hiromasa, Y., MENO, K. & Yoichi, A. Denaturation of the Bacillus stearothermophilus Dihydrolipoalnide Dehydrogenase in the Presence of Guanidine-HCl at Low Telnperature. *J. Fac. Agr., Kyushu Univ* **47**, 387-394 (2003).

8 Bhuiya, M. W. *et al.* Glutamate dehydrogenase from the aerobic hyperthermophilic archaeon Aeropyrum pernix K1: enzymatic characterization, identification of the encoding gene, and phylogenetic implications. *Extremophiles* **4**, 333-341 (2000).
